# Thermal Activation of High-Alumina Coal Gangue Auxiliary Cementitious Admixture: Thermal Transformation, Calcining Product Formation and Mechanical Properties

**DOI:** 10.3390/ma17020415

**Published:** 2024-01-14

**Authors:** Mingjun Zhang, Liang Li, Fan Yang, Shigang Zhang, He Zhang, Yongfu Zhu, Jian An

**Affiliations:** 1 Department of Materials Science and Engineering, Jilin University, Changchun 130025, China; zmj963258@163.com (M.Z.); zhang15166907043@163.com (S.Z.); zhanghe18686681322@163.com (H.Z.); 2 Jinneng Holding Coal Industry Group, Datong 037000, China; liangliang-2005@163.com (L.L.); yangfanjinneng88@163.com (F.Y.)

**Keywords:** high-alumina coal gangue, thermal activation, phase transformation, calcination product, mechanical properties

## Abstract

In this paper, a new preparation technology is developed to make high-alumina coal gangue (HACG) auxiliary cementitious admixture by calcining HACG–Ca(OH)_2_ (CH) mixture. HACG powders mixed with 20 wt.% CH were calcined within a temperature range of 600–900 °C, and the thermal transformation and mineral phase formation were analyzed. The hydration reaction between activated HACG–CH mixture and cement was also investigated. The results showed that HACG experienced a conventional transformation from kaolinite to metakaolin at 600 °C and finally to mullite at 900 °C, whereas CH underwent an unexpected transformation process from CH to CaO, then to CaCO_3_, and finally to CaO again. These substances’ states were associated with the dehydroxylation of CH, the chemical reaction between CaO and CO_2_ generating from the combustion of carbon in HACG, and the decomposition of CaCO_3_, respectively. It is the formation of a large amount of CaO above 800 °C that favors the formation of hydratable products containing Al_2_O_3_ in the calcining process and C-A-H gel in the hydration process. The mechanical properties of HACG–cement mortar specimens were measured, from which the optimal calcination temperature of 850 °C was determined. As compared with pure cement mortar specimens, the maximum 28-d flexural and compressive strengths of HACG–cement mortar specimens increased by 5.4% and 38.2%, respectively.

## 1. Introduction

As the largest producer and consumer of coal resources in the world, China has numerous coal mines across the country, of which yielded a coal output of 4.07 billion tons in 2021 [1]. Meanwhile, an increasingly serious and urgent problem facing the coal production enterprises is associated with the large amount of coal gangue (CG) discharged during the coal production process, since the annual output of CG amounts to hundreds of million tons and the accumulation of CG has reached 7 billion tons in China. CG is a type of rock with a low carbon content, and has long been regarded as solid waste in both China and other countries in the world [2,3]. Although a little part of CG has been reported to be utilized in various applications, such as road base material in road engineering, backfill material in coal mines and coarse aggregate in concrete production instead of natural stone material, the vast majority of CG has not been utilized effectively up to date, and still occupies lots of land for storage, which gives rise to a series of environmental pollution problems such as turbid air, muddy underground water and spontaneous combustion [4,5,6,7,8,9].

The chemical composition of CG varies enormously depending on the coal production region [10]. After removal of carbon in CG by the method of calcination, the remaining main components are Al_2_O_3_, SiO_2_ and CaO; their contents range widely, from 14.9 wt.% to 34.3 wt.%, 31.1 wt.% to 66.4 wt.% and 0.21 wt.% to 9.03 wt.%, respectively [11]. CG thus can be classified into three categories with respect of the content of Al_2_O_3_ or molar ratio of Al_2_O_3_ to SiO_2_ according to the classification of CG, i.e., high-alumina CG (HACG), claystone CG and sandstone CG [12]. Among them, HACG has the highest content of Al_2_O_3_, above 30 wt.%, and the highest molar ratio of Al_2_O_3_ to SiO_2_, ranging from 0.46 to 0.55, which means that the primary mineral constituent in HACG is kaolinite. HACG is primarily discharged in coal mines in North China, such as the Inner Mongolia Autonomous Region and Shanxi Province. The raw CG produced in coalfields in Datong city, Shanxi province, China, is reported to have a high level of carbon content, above 20 wt.%, but after calcining at 750 °C the remaining CG contains 32–38 wt.%Al_2_O_3_, 41–47 wt.%SiO_2_ and 0.05–0.15 wt.%CaO, respectively, which means that it is a typical type of HACG, with high carbon and low calcium oxide [13].

There have been many investigations on the applications of CGs in building materials [14,15,16,17]. As compared with ordinary Portland cement, both claystone CG and sandstone CG have a little higher content of Al_2_O_3_ and much higher content of SiO_2_, but a lower content of CaO. Therefore, these two types of CG are usually prepared as auxiliary cementitious admixture by the two following processing routes. The first route is to thermally activate CG at 700 °C and then to blend the activated CG auxiliary cementitious admixture in cement with a certain dosage for use. The second route is to increase calcium oxide content by the addition of minerals containing calcium element in CG, such as lime, limestone, gypsum and fluorite, and then to calcine CG with the minerals at a high temperature, above 1000 °C, for the formation of hydratable products such as 3CaO·SiO_2_ (C_3_S), 2CaO·SiO_2_ (C_2_S) and so on. The second route requires a high calcining temperature, since calcination at low temperatures of 500–900 °C was not found to have a significant effect on improving the activity of CG with the addition of minerals containing calcium [18,19,20,21].

Since HACG has a much higher content of Al_2_O_3_ and a much lower content of CaO than ordinary Portland cement, the above two commonly used processing routes have obvious drawbacks for making auxiliary cementitious admixture using HACG. In the case of the first route, the great amount of Al_2_O_3_ in HACG cannot be fully utilized to produce hydratable products because of the lack of an appropriate amount of CaO, while in the case of the second route, as the excessively high calcining temperature is required and consequently there is a low pozzolanic activity of mullite forms, this will consume too much energy and reduce the mechanical properties of HACG–cement mortar. Furthermore, there is an important problem concerning the selection of appropriate minerals containing CaO for HACG. To a certain extent, the mostly used minerals containing Ca element have little or big limitations. For example, limestone requires a high calcining temperature (about 900 °C) for decomposition to CaO and CO_2_, while lime can react with CO_2_ to form CaCO_3_ owing to a large amount of CO_2_ generating from the carbon combustion in HACG. These are major disadvantages that need to be overcome in the preparation of HACG auxiliary cementitious admixture. Therefore, a new preparation technology needs to be developed for making HACG auxiliary cementitious admixture by means of an effective material as a source of CaO and low-temperature activation.

The present paper is aimed at developing a new preparation technology to make HACG auxiliary cementitious admixture by adding Ca(OH)_2_ (CH) as the source of CaO and calcining at a low temperature (below 900 °C). The effect of calcining temperature on thermal transformation, evolution of the mineral product phase and surface structure of HACG was analyzed. The flexural and compressive strengths of HACG–cement mortar specimens were also measured, and from which the optimal calcining temperature was determined.

## 2. Experimental Details

### 2.1. Raw Materials

**Figure 1:**
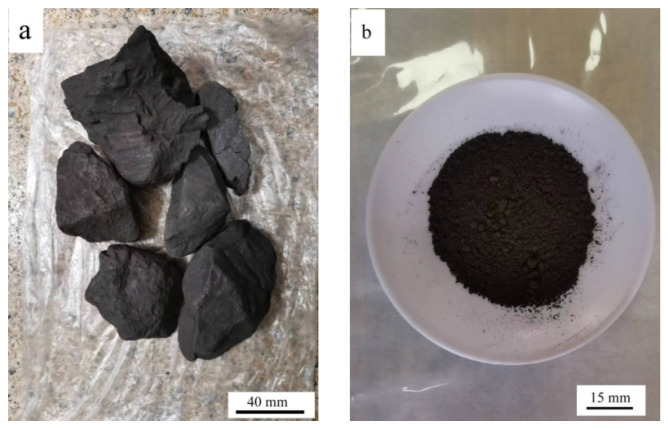
Raw HACG blocks (**a**) and ground powder (**b**).

**Figure 2:**
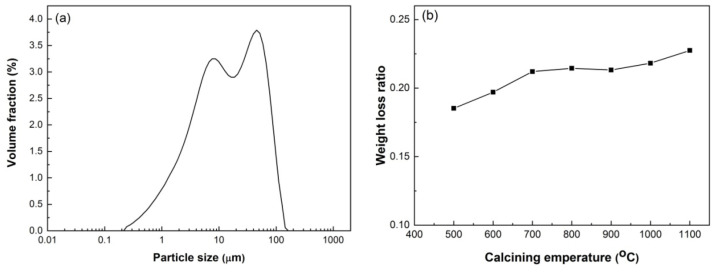
Particle size distribution of HACG powder (**a**) and weight loss ratio (**b**) after calcining at different temperatures.

HACG was supplied by Jinneng Holding Coal Industry Group in Datong city, Shanxi Province, China. The blocky HACG was washed, then crushed into pieces with sizes of 4–6 mm using a XPC-100 × 150 jaw crusher (Copenhagen, Denmark), and finally smashed into powder using a GJ-1A sealed sample preparation crusher (Shanghai, China). Raw HACG blocks and smashed powder are shown in Figure 1. The particle size of prepared HACG powder was measured using a Mastersizer 3000 laser particle size analyzer (Malvern, UK); the mean particle size (d_50_) of HACG powder was 14.9 μm. The HACG particle size distribution is shown in Figure 2a. The amount of carbon in the HACG powder was estimated by a weight loss ratio method, i.e., the weight loss ratio was obtained by the weight reduction after calcining 100 g HACG powder at a certain temperature for 2 h in a resistance furnace dividing the original weight. The weight loss ratios of as-received HACG at the calcining temperatures of 500–1100 °C are shown in Figure 2b. The weight loss ratio increased significantly with calcining temperature until 700 °C, and then entered a steady state. The weight loss ratios were 21.2% and 21.4% after calcining at 700 and 800 °C, respectively. This indicates that the raw HACG used in the present study contains a higher content of carbon than other types of CGs.

**Figure 3:**
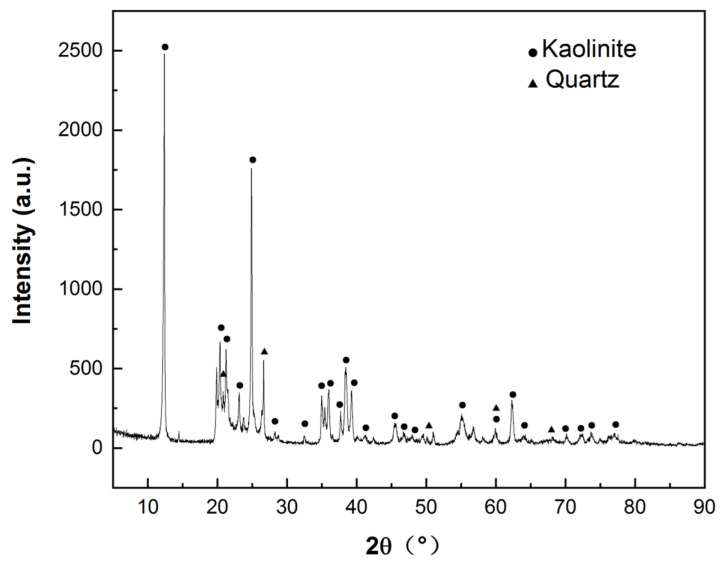
XRD pattern of as-received coal gangue.

The chemical composition of HACG powder calcined at 700 °C for 2 h was measured using the borate melting sample preparation method, as listed in Table 1. The calcined HACG has Al_2_O_3_ content as high as 45.01 wt.%, and a molar ratio of Al_2_O_3_ to SiO_2_ as high as 0.50, close to that of metakaolin, but has only 0.11 wt.% CaO. The CG used in the present study thus is a typical high-alumina and low-calcium oxide composition. The mineralogical phases in as-received coal gangue were determined by an A Rigaku D/MAX 2500PC X-ray diffractometer (XRD) (Tokyo, Japan), as shown in Figure 3, from which as-received HACG was identified to consist mainly of kaolinite (Al_2_O_3_·2SiO_2_·2H_2_O), with a high degree of crystallinity and a little amount of quartz. Ordinary Portland cement (P.O 42.5) was furnished by Yangchun Cement Co., Ltd. in Zhucheng City, China; its composition is also given in Table 1. ISO standard sand, produced by Xiamen ISO standard Sand Co., Ltd. in Xiamen City, China, was used for preparing mortar specimens.

### 2.2. Adding Calcium and Thermal Activation

In order to compensate for the lack of CaO in as-received HACG, CH (purity above 97 wt.%) powder with an average particle size of 22 μm was used to blend in HACG powder. CH powder accounted for 20 wt.% of the total weight of the HACG–CH mixture. Before thermal activation, HACG and CH powders were mixed thoroughly for 1 h in a V-type mixer. Thermal activation of HACG–CH mixtures was performed at the various temperatures of 600, 700, 800, 850 and 900 °C for 2 h in a resistance furnace, which finally yielded HACG auxiliary cementitious admixtures calcined at different temperatures. The thermal transformation and weight loss of the HACG–CH mixture were analyzed under Ar atmosphere and under air condition using a STA7300 thermogravimetric (TG)/differential thermal analyzer (DTA) (Tokyo, Japan), respectively. The calcining product phases and surface structures of the HACG auxiliary cementitious admixtures were analyzed using XRD and a VEGA3 TESCAN scanning electron microscope (SEM) (Brno, Czech Republic) equipped with an energy-dispersive X-ray spectrometer (EDS), respectively.

### 2.3. Hydration Products

HACG auxiliary cementitious admixtures calcined at different temperatures were mixed with cement thoroughly with a fixed ratio of 3 to 7, respectively. The HACG–cement mixtures were then added with water and stirred to prepare pastes. The ratio of HACG–cement mixture to water was 1 to 0.5. The pastes were poured into test tubes, and then cured in the sealed test tube at 20 °C. After being cured for 7 days, the prepared samples were taken out of the test tubes, smashed and ground into fine powders for identifying hydration products via the XRD technique.

### 2.4. Flexural and Compressive Strength

In order to evaluate the effect of calcining temperature on the flexural and compressive strength of HACG–cement mortar, HACG auxiliary cementitious admixtures calcined at different temperatures were blended thoroughly with Portland cement for 1 h in a V-type mixer. The HACG auxiliary cementitious admixture was used to replace 30% of Portland cement. HACG–cement mortar specimens were prepared according to the Chinese National Standard GB/T 17671-2021 [22]. HACG auxiliary cementitious admixture + Portland cement, sand and water were blended together in the weight proportion 1:3:0.5 to prepare regular square prism mortar specimens of 40 × 40 × 160 mm^3^. After curing in water at a room temperature of 20 °C for 7 days and 28 days, the prepared HACG–cement mortar specimens were used to test flexural and compressive strength *R_f_* and *R_c_*, as expressed by Equations (1) and (2), respectively.

(1) $$R_{f}=\frac{1.5F_{f}L}{b^{3}}$$

(2) $$R_{c}=\frac{F_{c}}{A}$$

where *F_f_* is the flexural fracture load, *L* is fulcrum spacing, i.e., 100 mm, *b* is the side length of the section, i.e., 40 mm, *F_c_* is the maximum compressive load, *A* is the compression area, i.e., 1600 mm^2^.

Small flakes with the fracture surfaces were obtained from 28-d flexural strength specimens. The fracture surfaces of small flakes were coated with gold to increase the conductivity, and then were observed using SEM and EDS for analyzing the fracture mode and chemical composition. The elemental mappings of O, Ca, C, Si, Al and S were conducted on the fracture surfaces to analyze the hydration state of HACG auxiliary cementitious admixture in HACG–cement mortar.

## 3. Results and Discussion

### 3.1. Thermal Transformation of HACG–CH Mixture

**Figure 4:**
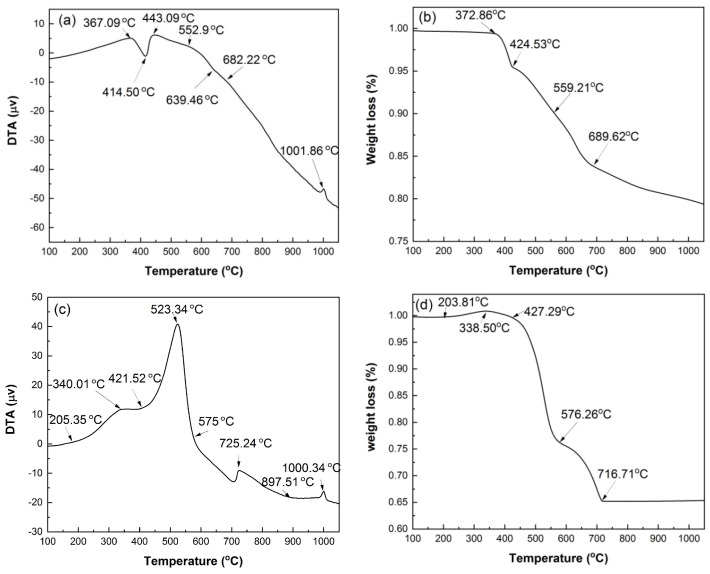
DTA and TG curves of mixed powders of coal gangue with CH addition, under Ar atmosphere and under air condition: (**a**) DTA in Ar, (**b**) TG in Ar, (**c**) DTA in air, (**d**) TG in air.

Figure 4 shows the DTA and TG curves of HACG powder mixed with CH obtained under Ar atmosphere and air conditions. The reason for conducting the measurement under Ar gas atmosphere is mainly to prohibit many influence factors, such as H_2_O and CO_2_ in air and the combustion of carbon in HACG, from being involved in the thermal transformation process, and thus the analysis of thermal transformation of the HACG–CH mixture would be simple and clear. Under Ar atmosphere, as seen from Figure 4a, there is an evident endothermic peak at 414.50 °C and a small exothermic peak at 1001.86 °C on the DTA curve. The former corresponds to the dehydroxylation of CH, and its onset and finish temperatures are 367.09 °C and 443.09 °C, respectively, while the latter represents the formation of mullite [23]. Moreover, there is an almost imperceptible endothermic peak between 552.97 and 682.22 °C, at 639.46 °C exactly, which derives from the dehydroxylation of kaolinite. This deduction was made based on the fact that raw coal gangue was found to be dehydrated to form metakaolin (Al_2_O_3_·2SiO_2_) in the temperature range of 555.73–689.90 °C by DTA analysis under Ar atmosphere (not shown here). It is noted that the dehydroxylation temperature for kaolinite in the studied HACG is much higher than that of CGs discharged in other regions in China. This might be attributed to a high degree of crystallinity in kaolinite for the studied HACG. The CGs, including kaolinite-type CG, discharged in Hebei Province and Jiangsu Province in China were reported to be dehydrated around a lower temperature between 510 °C and 550 °C [24,25]. Similarly, kaolinite in HACG produced in a Shuozhou coalfield in Shanxi Province, China, was also reported to undergo a dehydroxylation process in a higher temperature range of 550–700 °C [26]. The thermal transformations such as dehydroxylation of CH and kaolinite were directly reflected on the TG curve, as shown in Figure 4b. Almost no weight loss was observed before 372.86 °C, but a rapid weight loss occurred in the range of 372.86–424.53 °C due to dehydroxylation of CH; thereafter, weight loss proceeded moderately before 559.21 °C, and then became a little significant in the temperature range of 559.21–689.62 °C due to dehydroxylation of kaolinite, and finally turned to be mild until 1050 °C due to volatilization of some matters in HACG.

Under air condition, the thermal transformation and weight loss variation were more complicated than under Ar gas protection condition. As seen from Figure 4c, there are four exothermic peaks in the DTA curve. They are at 340.01 °C, 523.34 °C, 725.24 °C and 1000.34 °C, respectively. The first three are not observed on the DTA curve measured under Ar atmosphere. The first one resulted from the formation of a trace of CaCO_3_, i.e., CH reacted with CO_2_ in air to produce CaCO_3_ and H_2_O, as expressed by Equation (3). The second one originated from the combustion of carbon in HACG; namely, the burning of a large amount of carbon in HACG led to an intense and sharp exothermic peak. The third one was associated with the formation of CaCO_3_ due to a large amount of CO_2_ produced from the combustion of carbon in HACG and its reaction with the CaO generated from dehydroxylation of CH, as expressed by Equation (4). The fourth one corresponded to the formation of mullite. In addition, there were two endothermic peaks: the obvious one was at 421.52 °C, and the weak one was at 897.51 °C. The two endothermic peaks were attributed to the dehydroxylation of CH and the decomposition of CaCO_3_, respectively.

Ca(OH)_2_ + CO_2_ → CaCO_3_ + H_2_O (3)

CaO + CO_2_ → CaCO_3_ (4)

These thermal transformations were also reflected on the TG curve, as illustrated in Figure 4d. It was noted that a little weight gain occurred in the temperature range of 203.81–338.50 °C due to the formation of a trace of CaCO_3_ and H_2_O. The weight loss was mild in the temperature range of 338.50–427.29 °C owing to dehydroxylation of CH, and became more intense until 576.26 °C owing to carbon combustion in HACG; thereafter, it entered into a moderate-to-rapid stage until 716.77 °C owing to the effect of the dehydroxylation process of kaolinite and the opposite effect of CaCO_3_ formation. The evidences regarding CH dehydroxylation into CaO, and CaCO_3_ generating from the reaction between CaO and the carbon combustion-induced CO_2_, were further confirmed by the XRD and SEM analysis in Section 3.2 and Section 3.3.

### 3.2. Calcination Products of HACG–CH Mixtures

**Figure 5:**
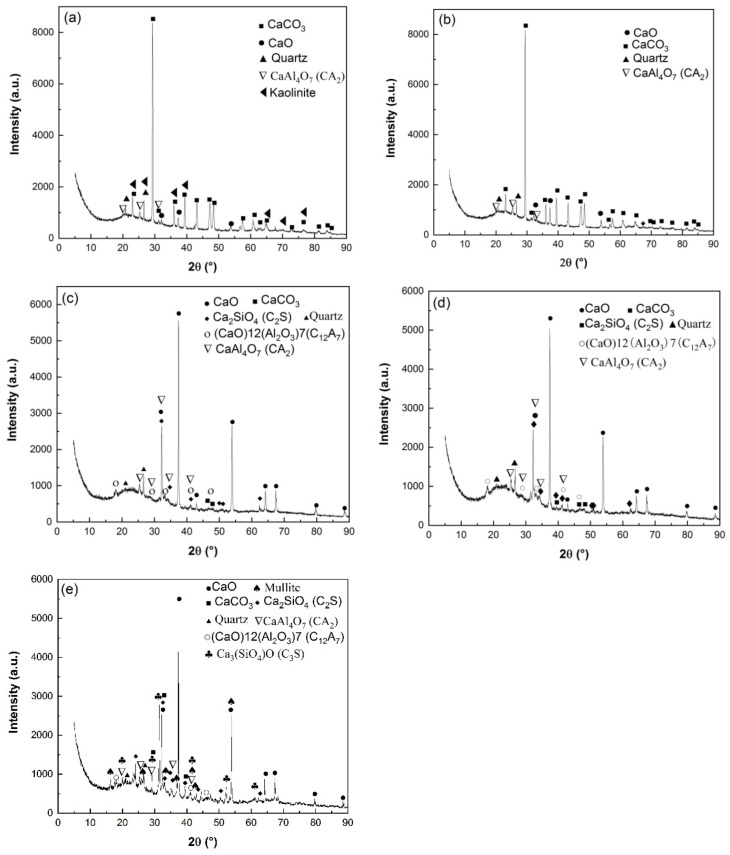
XRD patterns of HACG–CH mixtures calcined at different temperatures: (**a**) 600 °C, (**b**) 700 °C, (**c**) 800 °C, (**d**) 850 °C, (**e**) 900 °C.

The calcination products of HACG powders mixed with CH calcined at different temperatures was analyzed by XRD, as shown in Figure 5. At 600 °C, the typically strong diffraction peaks of kaolinite at 2 *θ* = 12.35° and 24.85° found in raw HACG disappeared, and a low-intensity hump emerged within 15° to 25°, suggesting that kaolinite began to transform into amorphous metakaolin (Figure 5a). Metakaolin is reported to have excellent pozzolanic properties for containing silica and alumina in an active form [27,28]. The combustion of a high amount of carbon in HACG could further promote the thermal decomposition of kaolinite and increase the reactivity of metakaolin. Yuan et al. [26] found that carbonaceous matter additives increased the decomposition rate of kaolinite and accelerated the structure transformation from scale-shaped lamellar to irregular and amorphous. Meanwhile, only a little amount of CaO and CaAl_4_O_7_ (CaO·2Al_2_O_3_ (CA_2_)) was found since their diffraction peaks were relatively weak. However, a great amount of CaCO_3_ was formed unexpectedly at this calcining temperature, since the strongest diffraction peak at 2 *θ* =29.36° belongs to CaCO_3_. Apparently, CH was totally dehydroxylated to form CaO at 600 °C, and a little part of CaO reacted with Al_2_O_3_ in metakaolin to form CA_2_ accordingly, as expressed by Equation (5). As for the formation of a large amount of CaCO_3_, C element in CaCO_3_ can only come from CO_2_ produced during the combustion of carbon in HACG, as a rapid and significant carbon combustion-induced weight loss occurred in the temperature range of 427.29–576.26 °C on the TG curve in air. Therefore, a large amount of CO_2_ reacted with the most part of CaO, resulting in the massive formation of CaCO_3_. At 700 °C, kaolinite was essentially transformed into metakaolin and the diffraction peaks of CaCO_3_ was still the most pronounced, while the other main constituent phases maintained their presence unchanged (Figure 5b). The massive formation of CaCO_3_ implies the useless consumption of quite a large amount of CaO generated from CH, which will result in insufficient CaO in the mixture for reacting with metakaolin during the calcining process and for reacting with metakaolin and water during the hydration process. Moreover, the incorporation of limestone (CaCO_3_) in Portland cement is usually required in many standards to be less than 5 wt.% because of its lack of cementitious and pozzolanic properties; otherwise, limestone leads to low strength, great porosity and great permeability of mortar and concrete [29,30].

CaO + Al_2_O_3_ → CaO·2Al_2_O_3_ (5)

As the temperature was increased to 800 °C, the diffraction peaks of CaCO_3_ almost disappeared due to decomposition, while CaO demonstrated the most prominent diffraction peaks (Figure 5c). The formation of a large amount of CaO is actually a turning point in the improvement of the reactivity of HACG. On the one hand, the transformation can facilitate the chemical reactions between CaO and SiO_2_ or Al_2_O_3_, namely the formation of hydratable mineral products such as Ca_2_SiO_4_ (2CaO·SiO (C_2_S)) and (CaO)12(Al_2_O_3_)7 (12CaO·7Al_2_O_3_(C_12_A_7_)), as expressed by Equations (6) and (7). On the other hand, a large amount of CaO can be transformed into quite a large amount of CH during the hydration process, which helps the hydration reaction among CH, active SiO_2_ or Al_2_O_3_ in metakaolin and H_2_O. Therefore, the presence of a large amount of CaO in the HACG–CH mixture could favor the enhancement in mechanical strength for HACG–cement mortar. At 850 °C, the main mineral products were almost the same as those formed at 800 °C, but the diffraction peaks of CA_2_, C_2_S and C_12_A_7_ became a little stronger (Figure 5d), indicating a little increase in their amounts. However, at the highest temperature, of 900 °C, the most significant change was the formation of crystallized mullite and (Ca_3_SiO_4_)O (3CaO·SiO (C_3_S)) (Figure 5e). Mullite was transformed from metakaolin, as expressed by Equation (8) [3], and C_3_S was formed by the reaction between CaO and SiO_2_, as expressed by Equation (9). C_3_S is well-known to be an effective hydratable product in cement, but mullite has almost no pozzolanic activity and consequently reduces the reactivity of CG [31,32,33]. Mullite has never been detected in HACG or other CGs by XRD analysis after being calcined at a temperature below 950 °C, according to the literatures available [24,25,27,32,33]. In the present study, the presence of mullite at 900 °C could be promoted by a series of reactions between CaO and Al_2_O_3_ or SiO_2_ in metakaolin. These reactions damage the metakaolin structure greatly, and accelerate the formation of mullite. Although the amount of mullite was little at this temperature, the presence of mullite in the HACG–CH mixture implies an increase in the crystallinity degree for the remained metakaolin, which could decrease the reactivity of metakaolin and bring about a negative effect on the mechanical properties of HACG–cement mortar.

2CaO + SiO_2_ → 2CaO·SiO (6)

CaO + Al_2_O_3_ → 12CaO·7Al_2_O_3_ (7)

3(Al_2_O_3_·2SiO_2_) → 3Al_2_O_3_·2SiO_2_ + 4SiO_2_ (8)

3CaO + SiO_2_ → 3CaO·SiO (9)

### 3.3. Surface Structures of HACG–CH Mixtures Calcined at Different Temperatures

**Figure 6:**
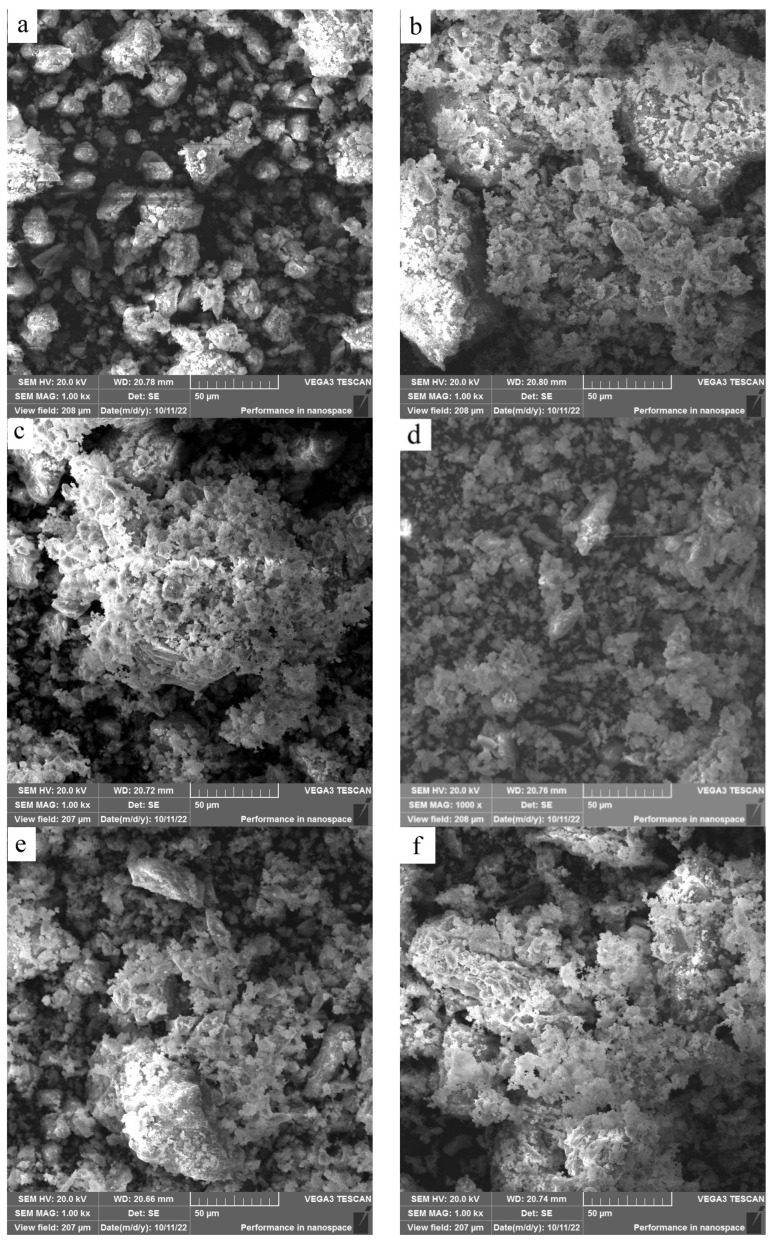
SEM images of the mixture of coal gangue and CH calcined at different temperatures: (**a**) original coal gangue powders, (**b**) 600 °C, (**c**) 700 °C, (**d**) 800 °C, (**e**) 850 °C, (**f**) 900 °C.

Figure 6 shows the morphologies of HACG powders mixed with CH calcined at different temperatures. As compared with original smashed HACG powder (Figure 6a), at 600 °C a lot of laminar products were formed on the HACG particles (Figure 6b). They appeared to be a weathered surface layer with wide spacing between each piece, exposing the HACG substrate underneath. Such a morphology could be brought about by dehydroxylation and the combustion of carbon in HACG or by the chemical reaction between HACG and CH. At 700 °C, most parts of the laminar products became tiny and dense, covering almost entire surfaces of the HACG particles, and the rest evolved into fine granules (Figure 6c). This morphological characteristic was associated with the formation of a large amount of CaCO_3_ on the original HACG particles, as identified by subsequent EDS analysis. At 800 °C, the surface products were essentially granular due to a large-scale decomposition of CaCO_3_ into CaO (Figure 6d), while at 850 °C some plate-like products occurred besides the granular particles, and they connected together (Figure 6e), which means that CaO reacted with metakaolin, producing more calcium aluminates and calcium silicates such as CA_2_, C_12_A_7_ and C_2_S, as revealed by subsequent EDS analysis. At the highest temperature, 900 °C, the morphology of surface products changed significantly: some fine powders appeared beside more connected plate-like products (Figure 6f), indicating that certain different reaction products were generated.

The chemical composition of surface products that formed on HACG powders mixed with CH calcined at different temperatures was analyzed by EDS point scanning. The resulting products were deduced to include CaO, CaCO_3_, metakaolin, CA_2_, C_2_S and C_12_A_7_ according to their respective compositional characteristics at locations indicated by numbers, as summarized in Table 2. The products that emerged at different temperatures were as follows: CaO, CaCO_3_ and metakaolin at 600 °C; CA_2_ at 700 °C; C_2_S at 800 °C; and C_12_A_7_ at 850 °C and 900 °C.

**Figure 7:**
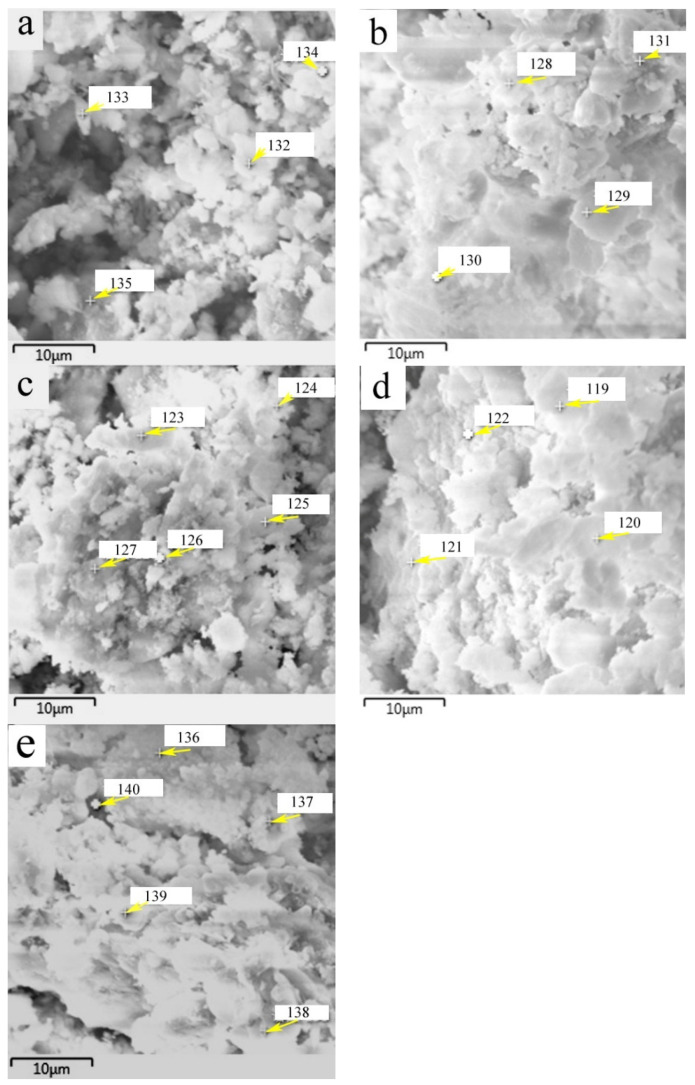
SEM images of the mixture of CG and CH calcined at different temperatures: (**a**) 600 °C, (**b**) 700 °C, (**c**) 800 °C, (**d**) 850 °C, (**e**) 900 °C.

**Figure 8:**
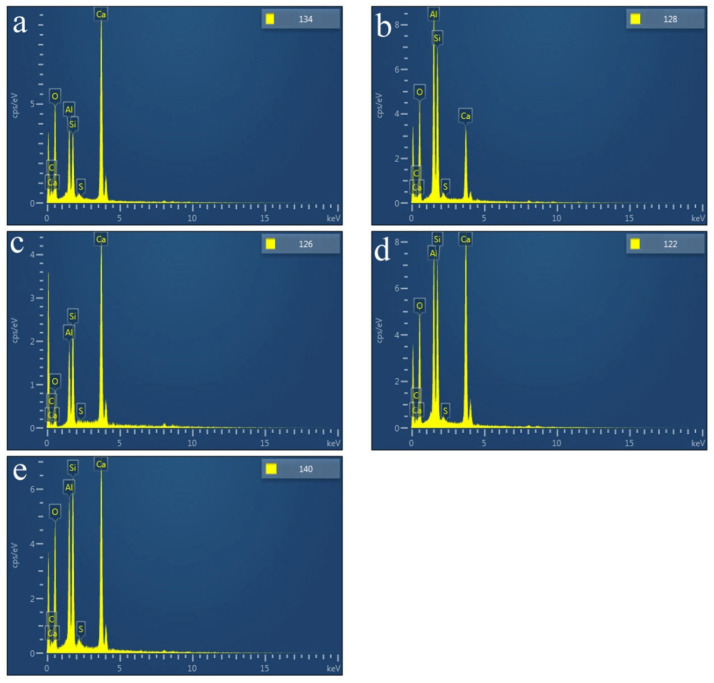
EDS patterns at certain indicated locations on the surfaces of mixtures of CG and OH calcined at different temperatures: (**a**) 600 °C, (**b**) 700 °C, (**c**) 800 °C, (**d**) 850 °C, (**e**) 900 °C.

SEM images of the HACG powders mixed with CH calcined at different temperatures are shown in Figure 7, in which EDS point scanning locations are indicated by numbers. EDS patterns at certain locations are shown in Figure 8. In order to identify calcining products as accurately as possible, two analysis methods were used. The first one was that if the composition at a location was close to that of a certain type of product, the product could be thus identified. For example, the product at location 135 had 22.35 wt.%Al, 22.61 wt.%Si, 53.78 wt.%O and only 1.14 wt.%Ca, suggesting that it originated from HACG. The contents of Al and Si elements were similar to the nominal composition of metakaolin: 21.26 wt.% Al and 22.05 wt.%Si. However, the content of O element was higher than 44.09 wt.%O in metakaolin. The product at location 123 was also identified as CaCO_3_ in such a method; the content of O and Ca elements are similar to the nominal 48 wt.%O and 40 wt.%Ca in CaCO_3_. The second one was taken by considering metakaolin composition involvement in the product reaction, which included two cases. In the first case, thin product layers could be formed on the metakaolin, such as CaCO_3_ and CaO. The composition of the product should be handled by reduction in the contents of O, Al and Si elements according to the proportions of O, Al and Si elements in metakaolin. That is, the content of O element is about twice the content of Al or Si elements in metakaolin. For instance, the product at location 129 had about 38 wt.%O and 36 wt.%Ca after reduction of 12 wt.%O according to the contents of 7 wt.%Al and 6 wt.%Si in metakaolin. The product with 38 wt.%O and 36 wt.%Ca roughly agreed with the normal chemical contents of O and Ca elements in CaCO_3_, i.e., 48 wt.%O and 40 wt.%Ca, and was thus deduced to be CaCO_3_ formed on the metakaolin substrate. In the second case, when Al_2_O_3_ or SiO_2_ in metakaolin reacted with CaO to form products such as CA_2_, C_12_A_7_ and C_2_S, the composition of products such as CA_2_, C_12_A_7_ and C_2_S should be evaluated by taking metakaolin as an ensemble using the following molecular formulas: CaO·2Al_2_O_3_·4SiO_2_, 12CaO·7Al_2_O_3_·14SiO_2_ and 2CaO·SiO·0.5Al_2_O_3_. The deducted products by EDS analysis in Table 2 were also previously identified from XRD patterns in Figure 5, but mullite and C_3_S were not found by EDS analysis, perhaps owing to the limited number of selected locations for analysis.

### 3.4. Hydration Products Generated from HACG Auxiliary Cementitious Admixture and Cement

**Figure 9:**
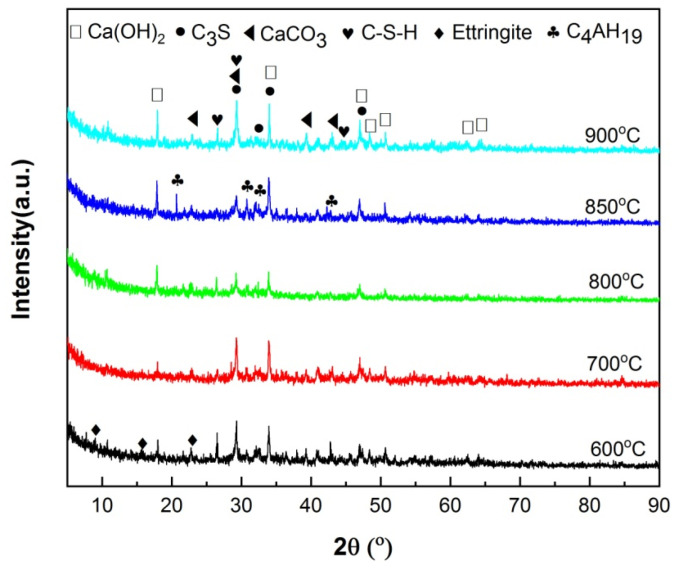
The XRD analysis of hydration products that originated from HACG auxiliary cementitious admixtures and cement.

The XRD patterns of hydrated HACG–cement powders are shown in Figure 9. The mineral phases were found to include C_3_S in cement, CaCO_3_ in calcined HACG auxiliary admixtures and several types of hydration products. The hydration products were CH, ettringite (Aft (3CaO·Al_2_O_3_·3CaSO_4_·32H_2_O)), C-S-H and 4CaO·Al_2_O_3_·32H_2_O (C_4_AH_19_), in which CH was originated from both cement and HACG auxiliary cementitious admixture; ettringite and C-S-H were produced from cement, but C_4_AH_19_ mainly came from HACG auxiliary cementitious admixture. The dependence of hydration products on the calcining temperature can be clearly observed in Figure 9. At the low calcining temperatures of 600 and 700 °C, the hydration products were ettringite, CH and C-S-H. However, as the calcining temperature rose above 700 °C, the diffraction peaks of CH became stronger than that at 600 and 700 °C. Apparently, it is associated with the formation of more CaO in HACG auxiliary cementitious admixture. The most noteworthy is that the diffraction peaks of C_4_AH_19_ emerge at the calcining temperature of 800 °C, and become more significant at 850 °C, suggesting that some of calcination products containing Al_2_O_3_ in HACG auxiliary cementitious admixture react with CH to form C_4_AH_19_. However, at the highest calcining temperature of 900 °C, those diffraction peaks from C_4_AH_19_ disappeared immediately. This could be due to the decrease in reactivity of metakaolin and the formation of mullite.

### 3.5. Flexural and Compressive Strength of HACG–Cement Mortar Specimens

**Figure 10:**
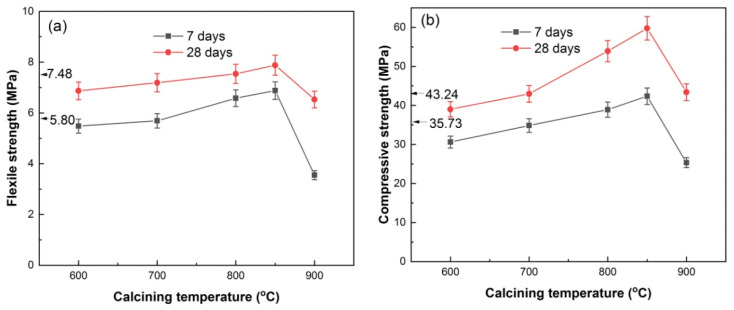
Flexural strength (**a**) and compressive strength (**b**) of HACG–cement mortar specimens vs. calcining temperature.

Figure 10 shows the flexural and compressive strength of HACG–cement mortar specimens. The pure cement mortar specimens had flexural strengths of 5.80 MPa and 7.48 MPa from 7 days curing and 28 days curing, respectively, and compressive strengths of 35.73 MPa and 43.24 MPa from 7 days curing and 28 days curing, respectively. With increasing the calcining temperature, the 7-d and 28-d flexural strengths of the HACG–cement mortar specimens increased until 850 °C, and then decreased rapidly at 900 °C (Figure 10a). At 800 and 850 °C, the HACG–cement mortar specimens had higher 7-d and 28-d flexural strengths than pure cement mortar specimens. Similarly, the 7-d and 28-d compressive strengths of the HACG–cement mortar specimens went up with increasing the calcining temperature and reached the maximum at 850 °C, and then went down considerably at 900 °C (Figure 10b). The maximal 28-d flexural and compressive strengths of the HACG–cement mortar specimens were increased by 5.4% and 38.2%, respectively, as compared with that of pure cement mortar specimens. As compared with the strength of mortar with the mixture of calcined CG and lime (CaO) as auxiliary cementitious material, the used HACG auxiliary cementitious material demonstrates an enhanced effect on the strength of mortar. Zhao et al. [20] prepared calcium added to calcined CG by mixing CG calcined at temperatures of 500–700 °C with 10% lime and 5% desulfurization gypsum, and found that the 7-d and 28-d flexural and compressive strengths of mortar specimens decreased with the content of calcium added to calcined CG. The reason for this may be due to the lack of calcining CG–lime mixture, so no hydratable products are generated from the chemical reaction between CaO and metakaolin.

The variation trend for flexural and compressive strengths with calcining temperature can be essentially elucidated on the basis of the XRD, EDS and hydration products results mentioned above. Even though hydratable CA_2_ was found to form at 600 and 700 °C, the mechanical properties of HACG–cement mortar were not increased significantly, since quite a large amount of undecomposed kaolinite and carbon still remained at 600 °C, and a massive formation of CaCO_3_ was originated from the combustion of carbon in HACG at 700 °C. These substances have low activity and could exert a negative effect on flexural and compressive strength. At higher calcining temperatures such as 800 and 850 °C, kaolinite transformed into active metakaolin thoroughly and a great amount of CaCO_3_ was discomposed into CaO, which promotes the reaction between metakaolin and CaO, and thus a variety of hydratable products such as C_2_S, CA_2_ and C_12_A_7_ were formed. These products can significantly increase the flexural and compressive strength of HACG–cement mortar during the hydration process. In addition, the excessive CaO can take part in the hydration reaction. However, at the highest temperature, of 900 °C, the amorphous metakaolin began to be crystallized and partly transformed into mullite, which weakened the activity of HACG auxiliary cement admixture and thus resulted in a considerable decrease in flexural and compressive strength.

### 3.6. Fracture Surfaces of HACG–Cement Mortar Specimens

**Figure 11:**
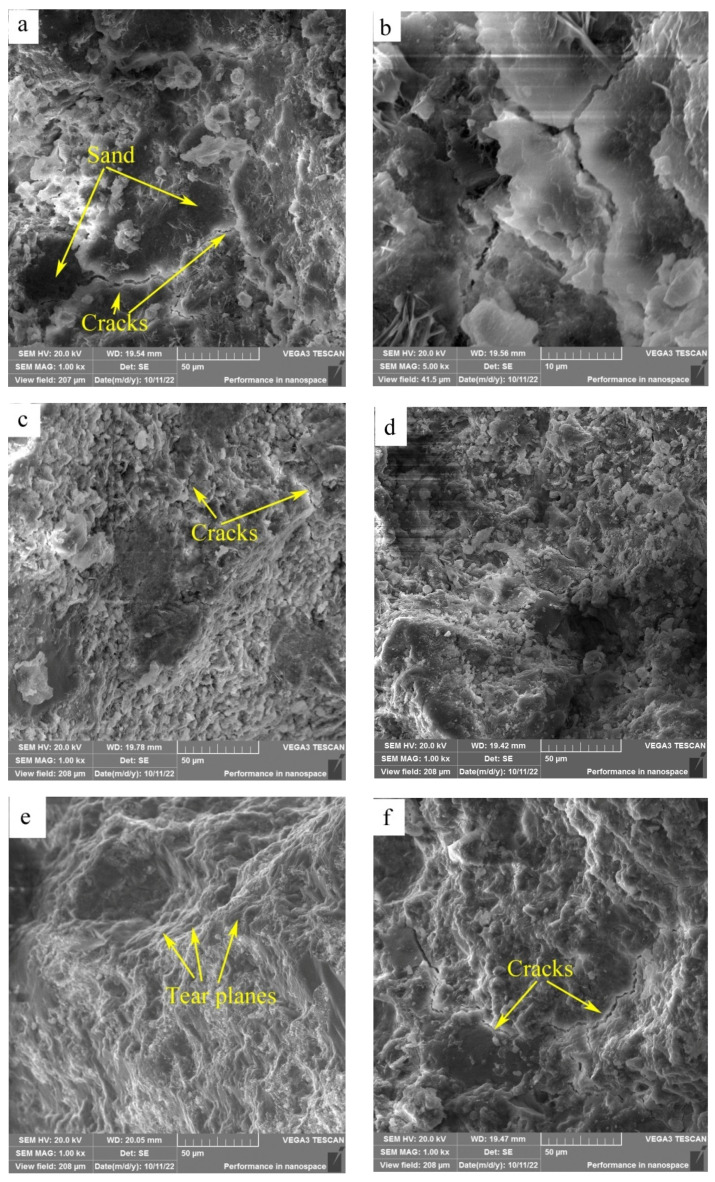
SEM images of fracture surfaces of mortar specimens with HACG cementitious admixtures calcined at different temperatures: (**a**) 600 °C, (**b**) 600 °C, (**c**) 700 °C, (**d**) 800 °C, (**e**) 850 °C, (**f**) 900 °C.

SEM images of the fracture surfaces of HACG–cement mortar specimens after 28-d flexural strength testing are shown in Figure 11. At the calcining temperature of 600 °C, the surface presented typical features of cleavage fracture, where the surface appeared flat and consisted of a number of large or small planes parallel to the fracture surface (Figure 11a). The large dark planes were parts of sand particles, as identified by EDS element mapping. Meanwhile, a few cracks were observed on the boundaries between sand particles and cementitious material, and also in the cementitious material region, as seen from the high-magnification photograph (Figure 11b), suggesting a weak bonding between the sand particle and cementitious material regions, and a low strength in the cementitious material region. At the calcining temperature of 700 °C, the surface looked a little rough and consisted of a large amount of lamellas and particles; meanwhile, cracks were also formed in the cementitious material region (Figure 11c), suggesting that constituents in the HACG auxiliary cementitious material were not well-hydrated and integrated. As the calcining temperature was increased to 800 °C, the surface consisted of dark sand particles and a grey cementitious material region (Figure 11d). No obvious cracks were found on the boundaries between sand and the cementitious material region besides a few microcracks in the cementitious material region, and there were still a few lamellas and particles in the cementitious material region, suggesting that constituents in the cement and HACG auxiliary cementitious admixture were still not bonded compactly. At the calcining temperature of 850 °C, the surface demonstrated a typical feature of tear fracture, where the surface consisted of a series of tear planes of different heights, and almost no cracks were observed on the surface (Figure 11e). Such a tear fracture mode indicates that every part in the HACG–cement mortar was bonded firmly. However, at the highest calcining temperature of 900 °C, the surface again showed the features of cleavage fracture. Meanwhile, a few large cracks were formed on the boundaries between sand and the cementitious material region, and were also found in the cementitious material region (Figure 11f), suggesting a weak bonding between sand and the cementitious material regions as well as among the constituents in the cementitious material. The variation in fracture surface morphology with calcining temperature agrees well with the trend of flexural and compressive strength of the HACG–cement mortar, i.e., when a good integrated boundary between sand particles and the cementitious material region is formed, and the constituents in the cementitious material are fully hydrated, the flexural and compressive strength of the HACG–cement mortar reach their respective maximum values. Thus, it is the reason for the mortar specimens using the HACG auxiliary cementitious admixture calcined at 850 °C to present the maximum mechanical properties.

The elemental mappings were conducted on the fracture surfaces by EDS (not all shown here). The chemical compositions of the surfaces are listed in Table 3. It is noted that the content of Ca element reaches a high level, above 20.1 wt.%, on the surfaces of mortar specimens with HACG auxiliary cementitious admixtures calcined at 800 and 850 °C, which is much higher than that on other mortar specimens’ surfaces. Meanwhile, the content of C element descends to a low level, below 13.4%, on the surfaces of mortar specimens with HACG auxiliary cementitious admixtures calcined at 800 and 850 °C, which is lower than that on other mortar specimens’ surfaces. The phenomenon of high-Ca and low-C in the mortar specimens with HACG auxiliary cementitious admixtures calcined at 800 and 850 °C indicates not only the significant burning loss of C in HACG, but also a large loss of CO_2_ due to the decomposition of CaCO_3_ into CaO and the subsequent formation of many hydratable products such as C_2_S, CA_2_ and C_12_A_7_ during the calcining process. On the other hand, the high level of CaO in the HACG auxiliary cementitious admixtures is conducive to the formation of more CH during the hydration process, so CH can react with H_2_O and active Al_2_O_3_ or SiO_2_ in metakaolin to form more hydration products such as C-A-H and C-S-H gel. Apparently, these advantages contribute greatly to the higher flexural and compressive strength shown by mortar specimens with HACG auxiliary cementitious admixtures calcined at 800 and 850 °C.

**Figure 12:**
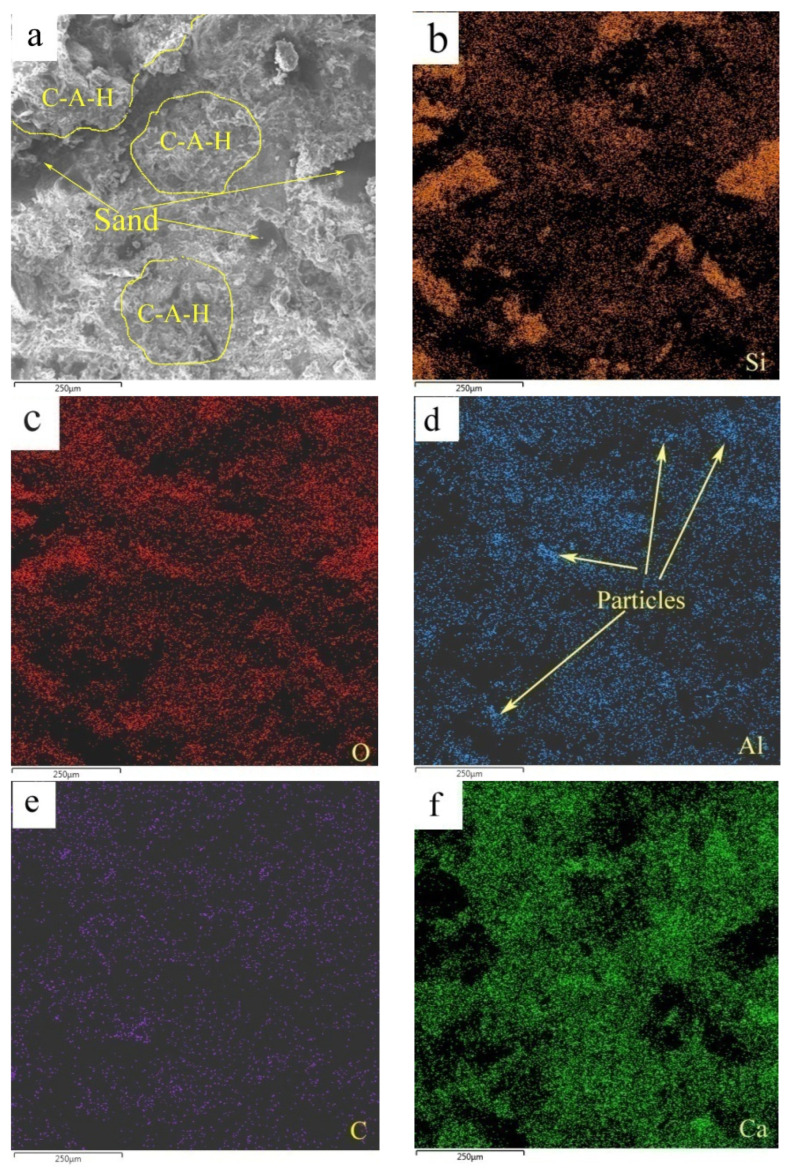
SEM image and elemental mappings (**b**–**f**) of fracture surface: (**a**) SEM image, (**b**) Si, (**c**) O, (**d**) Al, (**e**) C, (**f**) C.

**Figure 13:**
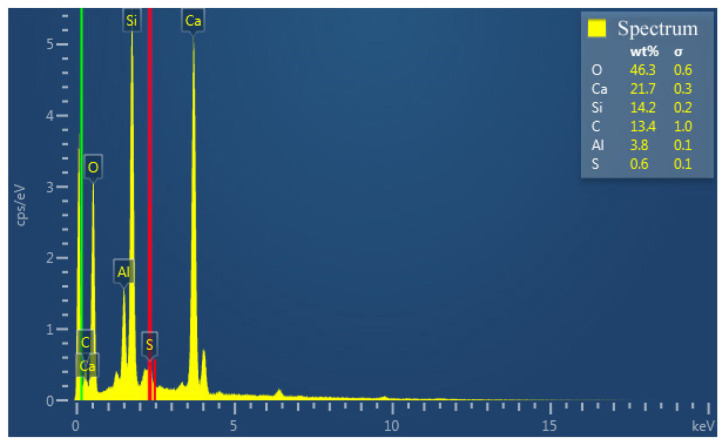
EDS pattern of fracture surface.

Figure 12 shows the elemental mappings of the mortar specimen fracture surface with HACG auxiliary cementitious admixture calcined at 850 °C. The corresponding EDS pattern of the fracture surface is shown in Figure 13. The dark flat areas indicated by yellow arrows on the SEM image were apparently occupied by sand particles (Figure 12a), which can be easily confirmed by the mappings for Si and O elements (Figure 12b,c). The mapping for Al and C elements told the locations of big and small calcined HACG particles (Figure 12d,e), as indicated by yellow arrows, since these particles exhibited the feature of high contents of Al and C elements in HACG. However, Ca element was found to be almost absent at these locations (Figure 12f), suggesting that the HACG particles were hardly involved in the hydration reaction, and made almost no contribution to the strengthening effect on mortar. Obviously, the number and size of HACG particles without involvement in the hydration reaction were less, and most Al-enriched areas were found to be distributed with Ca elements homogeneously, as indicated by yellow circles in Figure 12a. This proves that HACG auxiliary cementitious admixture calcined at 850 °C can be well-hydrated to form C-A-(S)-H gel.

## 4. Conclusions

This paper explored the formation mechanism of calcining products of HACG powders mixed with 20 wt.% CH and the mechanical properties of HACG auxiliary cementitious admixture–cement mortar specimens, and discovered that the calcining products and mechanical properties were greatly influenced by the calcining temperature. Specific conclusions drawn from this investigation are as follows:

1. The calcining temperature greatly influenced the transformation of the HACG–CH mixture and the chemical reaction between them. With increasing calcining temperature, HACG experienced the combustion of carbon and a transformation of kaolinite → metakaolin → mullite, while CH was dehydroxylated to form CaO at a temperature below 600 °C, and then a part of CaO underwent a transformation of CaO → CaCO_3_ → CaO. Meanwhile, several types of hydratable products were generated from the reaction between active metakaolin and CaO at different calcining temperatures.
2. A large amount of the CaO formed in the HACG–CH mixture calcined at 800 and 850 °C greatly contributed to the reaction between the active Al_2_O_3_ in the HACG–CH mixture and CH during the hydration process, in which the hydration product C_4_AH_19_ was formed.
3. With elevating the calcining temperature, the flexural and compressive strength of HACG–cement mortar specimens increased until 850 °C, and then decreased rapidly at 900 °C. As compared with pure cement mortar specimens, the maximum 28-d flexural and compressive strength of HACG–cement mortar specimens increased by 5.4% and 38.2%, respectively. The optimal calcination temperature for preparing HACG auxiliary cementitious admixture was thus 850 °C.
4. The finding of this investigation demonstrated that HACG powders with 20%CH addition could be utilized as an auxiliary cementitious admixture after being calcined at 850 °C, and that the flexural and compressive strength of HACG–cement mortar was much better than that of pure cement mortar, as HACG auxiliary cementitious admixture was used to replace 30% of Portland cement. This study expanded the application of HACG in auxiliary cementitious materials, but there are still many aspects such as long-term drying shrinkage and durability that need to be further investigated.

## Figures and Tables

**Table 1:** Chemical composition of coal gangue calcined at 700 °C for 2 h and cement (wt.%).

Material	SiO_2_	Al_2_O_3_	Fe_2_O_3_	CaO	MgO	SO_3_	K_2_O	Na_2_O	TiO_2_	P_2_O_5_	Loss
Coal gangue	52.60	45.01	0.21	0.11	0.06	-	0.12	0.04	0.97	0.03	0.35
Cement	21.04	6.03	3.62	63.85	2.64	2.21	-	-	-	-	0.21

**Table 2:** Chemical composition and product at indicated locations.

Temperature (°C)	O	Al	Si	S	Ca	Location	Product
600	45.16	13.43	11.66	0.06	29.21	132	CaO
	56.17	7.19	6.70	0.00	29.93	134	CaCO_3_
	53.78	22.35	22.61	0.01	1.41	135	Metakaolin
700	49.80	19.46	18.03	0.00	12.69	128	CA_2_
	50.42	7.36	5.87	0.00	36.32	129	CaCO_3_
	58.00	17.30	17.79	0.00	6.78	131	Metakaolin + CaCO_3_
800	51.84	2.51	2.11	0.31	43.23	123	CaCO_3_
	11.81	11.70	11.53	0.02	64.92	125	CaO
	36.85	10.25	13.49	0.17	39.23	126	C_2_S
	45.67	4.42	4.15	0.31	45.45	127	CaO
850	45.10	7.43	6.93	0.12	39.97	119	CaO
	45.10	9.68	9.23	0.12	35.87	120	CaO
	52.03	20.27	20.45	0.00	7.25	121	CA_2_
	49.87	13.15	13.00	0.00	23.97	122	C_12_A_7_
900	55.13	2.30	2.26	0.24	33.60	136	CaCO_3_
	45.70	9.12	9.75	0.09	35.43	137	CaO
	40.56	23.67	26.11	0.00	9.65	138	CA_2_
	51.56	11.18	13.47	0.00	23.74	140	C_12_A_7_

**Table 3:** Chemical compositions of fracture surfaces using CG admixtures calcined at different temperatures.

Temperature (°C)	O	Ca	C	Si	Al	S
600	50.2	18.2	14.2	12.8	4.1	0.6
700	47.8	13.9	15.8	20.1	2.1	0.3
800	44.9	20.1	12.9	18.0	3.7	0.5
850	46.3	21.7	13.4	14.2	3.8	0.6
900	48.9	16.7	16.8	13.4	3.5	0.7

## Data Availability

Data are contained within the article.
